# Unilateral Duane Retraction Syndrome Associated with Unilateral Congenital Cataract

**DOI:** 10.18502/jovr.v15i1.5952

**Published:** 2020-02-02

**Authors:** Majid Farvardin, Alireza Bolkheir

**Affiliations:** ^1^Poostchi Ophthalmology Research Center, Shiraz University of Medical Sciences, Shiraz, Iran

**Keywords:** Congenital Cataract, Duane Retraction Syndrome

## Abstract

**Purpose:**

To report unilateral congenital cataract in a case of ipsilateral Duane retraction syndrome.

**Case Report:**

In this case, we present a six year old girl who was referred with ocular deviation. She had a history of congenital cataract surgery in the left eye at the age of two years. The subject had no associated systemic disease, developmental delay, or positive family history. She was finally diagnosed as having Duane retraction syndrome in the same eye.

**Conclusion:**

Duane retraction syndrome can be associated with congenital cataract due to the matching time of gestational development of the lens to that of ocular and non-ocular anomalies associated with Duane syndrome. As both of these disorders are rare, coincidence of both in the same person and the same eye by chance is a very remote possibility.

##  INTRODUCTION

Duane retraction syndrome is a rare condition, for which mechanical, anatomical, and innervational disorders of the extraocular muscles are suggested as the etiologies.^[1]^ As a teratogenic congenital cranial innervational disorder at 4^th^-8^th^ weeks of gestation, Duane retraction syndrome has a 12% chance of association with other congenital ocular anomalies.^[2]^ In this article, we present a six year old girl who was referred with ocular deviation. She had a history of congenital cataract surgery in the left eye at the age of two years. She had no associated systemic disease or developmental delay and family history was unremarkable. She was finally diagnosed as having Duane retraction syndrome in the left eye.

##  CASE REPORT

A six year old girl was referred to the Strabismus and Pediatric Ophthalmology Service at the Shahid Mottahhari Clinic (a referral eye clinic in the south of Iran) due to outward deviation and decreased visual acuity in her left eye.

She was the first child in her family, born by cesarean section because of her mother's uncontrolled gestational diabetes mellitus. Her birth weight was 3.2 kg. At the age of two years, she underwent lensectomy and posterior intraocular lens implantation due to a congenital cataract in the left eye. Her family history was negative for congenital cataract and ocular deviations and she had no neurodevelopmental delay.

**Figure 1:**
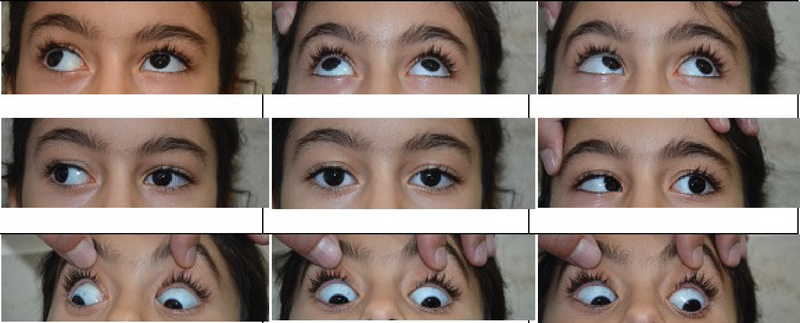
Exotropic Duane retraction syndrome, left eye. Top row, upshoot upon adduction in the left eye. Middle row, exotropia in primary position, limitation of abduction and adduction with retraction of the globe on adduction, all in the left eye. Bottom row, downshoots upon attempted adduction in the left eye.

The ophthalmologic examination revealed the best corrected visual acuity (BCVA) as 20/25 in the right eye and 20/300 in the left eye. Cycloplegic refraction was +0.25–0.25*125 in the right eye and +1.5–2.00*170 in the left eye. Intraocular pressure, measured by air puff tonometry, was 17- and 13-mmHg in the right and left eyes, respectively. She had a left exotropia of 8 prism diopters in primary position. Ocular motility examination showed limitation in adduction and abduction with narrowing of the palpebral fissure and downshoot and upshoot upon attempted adduction in the left eye which was consistent with a left Duane retraction syndrome. Vertical ductions were within normal limits in both eyes [Figure 1]. An examination of the anterior segment revealed a posterior chamber intraocular lens in the capsular bag with a posterior capsular opacity in the left eye. The result of dilated fundus examination was unremarkable in both eyes. Due to the small angle deviation and amblyopia in the left eye, we recommended right eye patching (4 hours per day) and follow up.

##  DISCUSSION

Congenital cataract is the most common treatable reason for blindness in children, which occurs in 1–15:10,000 children.^[3]^ Only 20% of the patients have a positive family history and 3–15% have some types of infectious etiology.^[3,4,5]^ It may be associated with other ocular or systemic abnormalities in half of the patients.^[4]^ Two-third of the patients have bilateral cataract, which includes more than 90% of the hereditary cases, but half of the sporadic cases are unilateral.^[5]^ Our patient had a congenital cataract only in the left eye and no family history of congenital cataract. Her disease was diagnosed at the age of two years, but at the time of diagnosis, no concomitant ocular or systemic disorder was noticed. She underwent a cataract surgery in the left eye.

The child described here had not been investigated for concomitant abnormalities until she was referred to us, when exotropia in the primary gaze, limitation in adduction and abduction, narrowing of the palpebral fissure, and downshoots and upshoots on attempted adduction (all in her left eye) led us to the diagnosis of Duane retraction syndrome. Duane retraction syndrome is expected to be diagnosed early, because the symptoms are usually quite evident. Nevertheless, some cases have been reported with delayed diagnosis.^[6]^

The mechanical etiology of Duane retraction syndrome is suggested to be fibrosis of the extraocular muscles and abnormal insertions.^[7]^ This syndrome has also been classified as a cranial dysinnervation due to the paradoxical innervation of the rectus muscles along with central nervous system anomalies.^[1]^ Duane retraction syndrome accounts for 1–4% of all strabismus patients and its incidence had been estimated as 0.04% in general population.^[6,8]^ According to DeRespinis and colleagues, the main ocular and non-ocular abnormalities associated with Duane retraction syndrome occur during the 4^th^–8^th^ gestational weeks, while the cranial nerves and nuclei (3, 4, and 6) develop during the 5^th^–8^th^ and extraocular muscle innervation during the 4^th^–6^th^ gestational weeks.^[1]^ On the other hand, the lens forms during the invagination of the surface ectoderm overlying the optic vesicle at approximately the 4^th^ week of gestation. The embryonic nucleus develops by the 6^th^ week of gestation.^[5]^ Thus, the time of gestational development of the lens matches that of the ocular and non-ocular anomalies associated with Duane retraction syndrome, and the same teratogenic factor may lead to both disorders. As both of these disorders are rare, coincidence of them by chance in the same person and the same eye is a very remote possibility.

Several non-ocular anomalies such as deafness, musculoskeletal disorders, cardiac defects, and other disorders have been associated with Duane retraction syndrome. The majority of cases are non-syndromic and sporadic, mainly occurring in female patients and in the left eyes.^[1,2,9]^ Although our patient had no developmental delay or systemic disorder, a number of cases may be associated with neurodevelopmental delay,^[6]^ and some show inheritance and mutations.^[10]^ Mohan and colleagues reported 12% associated ocular abnormalities among 331 patients with Duane retraction syndrome (291 unilateral and 40 bilateral cases). They reported the following findings in patients with unilateral Duane retraction syndrome: congenital ptosis (8%), congenital nasolacrimal duct obstruction, conjunctival dermolipoma, and nystagmus (0.6% each), orbital dermoid cyst, congenital ptosis with Marcus Gunn phenomenon, distichiasis, multiple conjunctival nevi, bilateral congenital cataract, and situs inversus of the disc and nasal crescent (0.3% each). In the patients with bilateral Duane retraction syndrome, they found congenital ptosis (5%), congenital ptosis with Marcus Gunn phenomenon, bilateral microcornea, choroidal coloboma, and nystagmus (2.5% each).^[2]^ In their case series, they reported one patient with simultaneous unilateral Duane syndrome and bilateral congenital cataract.^[2]^ Ahluwalia and colleagues reported another case of congenital cataract in their study of 20 patients with Duane retraction syndrome.^[8]^ DeRespinis *et al* described congenital cataract as a less frequent ocular finding in Duane retraction syndrome.^[1]^ An association of Duane retraction syndrome and lenticonus has also been reported in two patients from Canada, and China.^[11,12]^

Several classifications have been suggested for Duane retraction syndrome (types I, II, III) and the subtypes are suggested to stem from the same pathogenesis.^[1]^ The most commonly used classification is categorizing patients as having esotropia, exotropia, or orthotropia.^[1,9]^ In our case, the patient experienced exotropic Duane retraction syndrome.

As the overall prevalence of Duane retraction syndrome is low, it is important to know the main symptoms and signs of this syndrome and consider the differential diagnoses including the pseudo Duane retraction syndrome. Considering the gestational age of Duane retraction syndrome development, ophthalmologists should be aware of any possible ocular or systemic anomalies that may occur at the same gestational age. Familial inheritance is observed in 10% of cases with Duane retraction syndrome;^[1]^ therefore, ophthalmologists should examine the siblings and other family members for this disorder. Report of new cases can improve the knowledge of ophthalmologists, ensure the timely treatment, and prevent further complications.

##  Financial Support and Sponsorship

Nil.

##  Conflicts of Interest

There are no conflicts of interest.
